# Dermoscopy of Acquired Perforating Dermatoses: A Case Series and Review of the Literature

**DOI:** 10.3390/life15121786

**Published:** 2025-11-21

**Authors:** Maria-Myrto Papadopoulou, Emmanouil Karampinis, Dimitrios Sgouros, Styliani Sakellaropoulou, Elizabeth Lazaridou, Aimilios Lallas, Zoe Apalla

**Affiliations:** 1 Second Dermatology Department, School of Health Sciences, Aristotle University of Thessaloniki, 54124 Thessaloniki, Greece; emankarampinis@gmail.com (E.K.); stellasakel@gmail.com (S.S.); bethlaz@auth.gr (E.L.); zoimd@yahoo.gr (Z.A.); 2 Department of Internal Medicine, General Hospital of Karditsa, 43131 Karditsa, Greece; 3 Department of Dermatology, Faculty of Medicine, School of Health Sciences, University General Hospital of Larissa, University of Thessaly, 41110 Larissa, Greece; 4 Second Department of Dermatology and Venereology, Medical School, National and Kapodistrian University of Athens, “Attikon” General University Hospital, 12462 Athens, Greece; disgo79@gmail.com; 5 Department of Dermatology, 424 Military Training Hospital, 56429 Thessaloniki, Greece; 6 First Dermatology Department, School of Health Sciences, Aristotle University of Thessaloniki, 54124 Thessaloniki, Greece; emlallas@gmail.com

**Keywords:** acquired perforating dermatoses, reactive perforating collagenosis, Kyrle’s disease, perforating folliculitis, elastosis perforans serpiginosa, dermoscopy, transepidermal elimination, concentric pattern

## Abstract

Acquired perforating dermatoses (APD) represent a group of papulonodular skin disorders characterized by transepidermal elimination of dermal components, most frequently arising in patients with poorly controlled chronic systemic conditions such as diabetes mellitus (DM) and chronic renal failure (CRF). The four classical subtypes include acquired reactive perforating collagenosis (RPC), Kyrle’s disease (KD), elastosis perforans serpiginosa (EPS), and perforating folliculitis (PF). Owing to their rarity and the often-complex comorbidities of affected individuals, accurate diagnosis of APD may be challenging. In this context, dermoscopy has emerged as a valuable noninvasive tool that enhances diagnostic accuracy and supports clinical decision-making. This study aimed to characterize the dermoscopic features of APD through a case series and subsequent literature review. We present clinical and dermoscopic findings from a case series of 10 patients with APD followed by a literature review of 17 published case reports and 2 case series. The predominant dermoscopic pattern comprised a central yellow-to-brown structureless area, a surrounding white rim or a broader white structureless area with or without scaling, and an outer erythematous area containing dotted or hairpin vessels. Variations in these features appeared to reflect different stages of lesion evolution. The findings reinforce dermoscopy as a useful adjunct for the recognition, characterization, and monitoring of APD, providing additional insights into disease progression and contributing to improved diagnostic accuracy and clinical management.

## 1. Introduction

Perforating dermatoses (PD) constitute a group of rare papulonodular cutaneous disorders characterized by the transepidermal elimination of connective tissue components. Such manifestations may serve as the primary indication of either familial or acquired systemic conditions. Familial primary perforating disorders typically present during childhood, with limited documented cases in the literature. Conversely, perforating dermatoses observed in adult patients with systemic diseases, including diabetes mellitus (DM) and chronic renal failure (CRF), have been more commonly acknowledged, leading to the introduction of the term “acquired perforating dermatoses” (APD) [1].

Four classical forms of primary acquired perforating dermatoses have been identified: reactive perforating collagenosis (RPC), Kyrle’s disease (KD), perforating folliculitis (PF), and elastosis perforans serpiginosa (EPS). Acquired reactive perforating collagenosis involves the transepidermal elimination of collagen fibers, while elastosis perforans serpiginosa entails the expulsion of elastic fibers. Kyrle’s disease is characterized by the transepidermal elimination of abnormal keratin, and perforating folliculitis involves the content of the follicle. Secondary forms, in which transepidermal elimination occurs secondary to another skin disease, encompass perforating granuloma annulare, calcinosis cutis, necrobiosis lipoidica, chondrodermatitis nodularis helicis and keratoacanthoma [2,3].

Both genders are almost equally affected by APD, which typically manifest during the fifth-sixth decade of life [4]. Typically, patients exhibit hyperkeratotic papules on the extensor areas of extremities, often over regions of superficial trauma, while Koebner phenomenon may be present. The lesions are highly pruritic and may evolve into sizable, umbilicated papules, nodules or plaques with a central keratotic plug and an erythematous border, exhibiting a relapsing and remitting course over the patient’s lifetime [3].

Although APD have been linked to a variety of systemic conditions—including chronic renal failure, diabetes mellitus, thyroid dysfunction, hypertension, hepatic disease, and malignancies—the underlying pathogenesis remains unclear. Microtrauma from scratching and chronic pruritus have been suggested as a contributing factor in lesion development [5,6]. In this context, the primary goal of APD therapy is to control the underlying disease and to alleviate pruritus [4].

The uncommon occurrence of APD makes accurate diagnosis challenging, highlighting the need for improved diagnostic strategies. Dermoscopy represents a rapid and noninvasive diagnostic technique that can be employed as an adjunct to routine clinical examination, providing enhanced visualization and additional diagnostic information regarding cutaneous disorders. In the context of perforating dermatoses, dermoscopy may enhance diagnostic accuracy by revealing specific vascular, follicular, and structural patterns that assist in distinguishing clinically similar entities. Its application in the evaluation of APD has proven particularly useful, as APD often presents with nonspecific clinical features that overlap with other pruritic and hyperkeratotic dermatoses, such as prurigo nodularis (PN) and lichen planus [7,8].

Given the diagnostic challenges posed by APD and the growing recognition of dermoscopy as a pivotal tool in dermatologic assessment, a systematic appraisal of its dermoscopic hallmarks is warranted. The present study reports a case series of patients diagnosed with APD, accompanied by a comprehensive literature review focused on dermoscopic characteristics associated with this condition. The aim is to delineate the key dermoscopic features of APD and highlight their diagnostic relevance. Then, our findings were placed within the existing body of evidence in order to support a more standardized and objective approach to the diagnosis and differentiation of APD. To our knowledge, this is the first literature review study comprehensively addressing the dermoscopic features of APD.

## 2. Materials and Methods

We conducted a retrospective, descriptive study aimed at characterizing the dermoscopic features of APD and identifying specific diagnostic clues that may facilitate accurate, non-invasive diagnosis. The study included 10 patients with histopathologically confirmed APD who presented to our outpatient dermatology clinic in Thessaloniki, Greece. In addition, data from 24 previously reported cases retrieved from the literature were analyzed for comparison. All patients provided written informed consent for inclusion in this case series and for the open-access publication of clinical and dermoscopic images.

The comprehensive literature review was performed using the PubMed database from its inception until September 2025. The following keywords and their combinations were used: “Perforating Dermatoses,” “Acquired Perforating Dermatoses,” “Acquired Reactive Perforating Collagenosis,” “Kyrle’s Disease,” “Elastosis Perforans Serpiginosa,” “Perforating Folliculitis,” AND “Dermoscopy.” The initial search yielded 41 results. After screening titles, abstracts, and references, we included studies that met the following inclusion criteria: (1) case report or case series design, (2) complete clinical and dermoscopic documentation of all cases, (3) human subjects, and (4) publication in English. According to these criteria, 19 studies were selected for inclusion, comprising 2 case series and 17 case reports.

## 3. Results

### 3.1. Case Series

**Figure 1:**
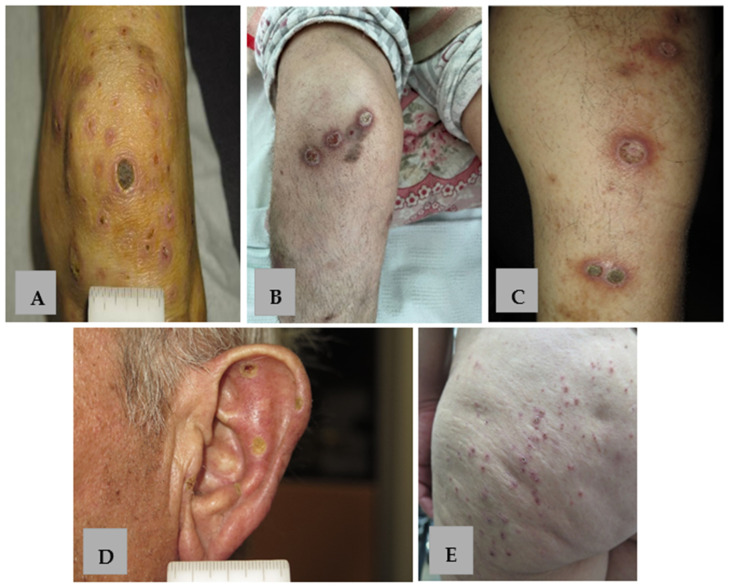
Clinical presentation of APD. (**A**–**C**) Keratotic papules, nodules and plaques with erythematous borders on the extensor surface of the lower extremities in patients 1, 5, 8 with KD. (**D**) Umbilicated keratotic papules with yellow-to-green crusts on the external surface of the ear in patient 9 with RPC. (**E**) Keratotic follicular papules on the gluteal region in patient 6 with PF.

**Figure 2:**
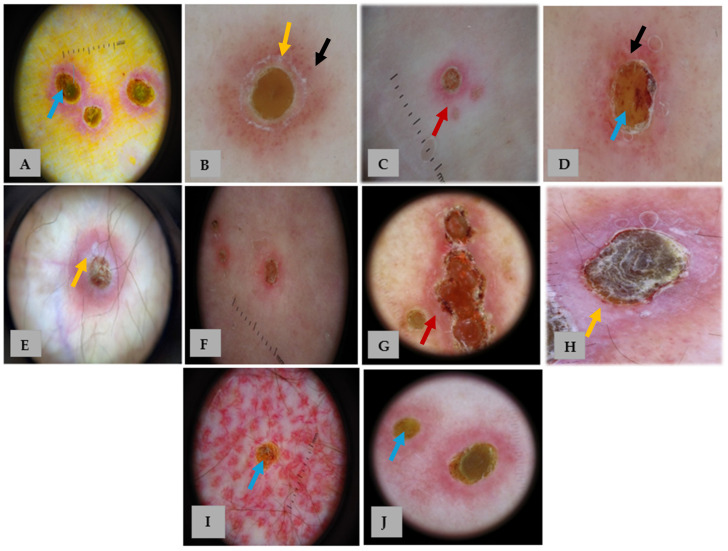
Dermoscopic images of our 10 patients with APD (**A**–**J**). Panels (**A**–**J**) correspond to patients 1–10, respectively. Dermoscopy shows a characteristic three-zoned concentric pattern: a central yellow-to-brown structureless area (blue arrows), a middle white rim (yellow arrows) with or without scale, and an outer erythematous structureless area (red arrows) with occasional dotted vessels and hairpin (black arrows). The detailed distribution of dermoscopic features is summarized in Table 1.

We conducted a clinical and dermoscopic evaluation of 10 patients with the histopathological diagnosis of PD (Figure 1 and Figure 2). Within this case series, 3 patients were identified with RPC, 4 with KD, and 3 with PF. None of the patients in our sample were diagnosed with EPS. For each case, we documented the age, sex, disease distribution, symptoms, the presence of concomitant diseases (if any), and the results of the clinical and dermoscopic assessment (Table 1).

**Table 1:** Clinical, dermoscopic and histological findings in our case series of 10 patients.

Patient No.	Age (Years)	Sex	Distribution	Symptom	Concomitant Diseases	Clinical Findings	Dermoscopic Findings	Histopathological Diagnosis
1	53	female	lower extremities	pruritus	recent scabies infection, CRF	keratotic nodules, papules and plaques with an erythematous border	central yellow-to-brown structureless area surrounded by a white rim and a pink structureless area with dotted vessels	KD
2	53	female	upper and lower extremities	pruritus	-	skin-colored to erythematous papules with central keratotic plug and an erythematous border	central yellow-to-brown structureless area with a rim of white scales surrounded by a pink structureless area with dotted vessels	PF
3	65	female	trunk, lower extremities, and buttocks	pruritus	microcellular lung cancer treated with atezolizumab, carboplatin and etoposide	keratotic papules with an erythematous border	central yellow-to-brown structureless area with a rim of white scales surrounded by a pink structureless area with dotted vessels	RPC
4	51	female	lower extremities	pruritus	uterine cancer/staphylococcal bacteremia/pericarditis	skin-colored to erythematous papules with central keratotic plug and an erythematous border	central yellow-to-brown structureless area surrounded by a rim of white scales and a red structureless area with dotted vessels, hairpin vessels and blood spots	PF
5	46	female	lower extremities	pruritus, pain	myasthenia gravis treated with azathioprine and corticosteroids	crateriform papules and plaques with erythematous border, ulcerations	central yellow-to-brown structureless area surrounded by a white rim and a peripheral pink structureless area	KD
6	59	female	trunk, buttocks	pruritus	-	skin-colored to erythematous papules with central keratotic plug and an erythematous border	central yellow-to-brown structureless area with blood spots surrounded by a rim of white scale and a pink structureless area	PF
7	90	female	shoulders, chest	pruritus	-	keratotic papules and plaques with an erythematous border	central hemorrhagic structureless area surrounded by white scale, blood spots and a red structureless area	KD
8	73	male	upper and lower extremities	pruritus	-	crateriform papules and plaques with an erythematous border, atrophic scars	central yellow-to-brown structureless area with white clods and blood spots surrounded by a white irregular rim with scale and a pink structureless area	KD
9	69	male	ears, upper extremities	pruritus	-	keratotic papules, yellow-to-green crusts	yellow-to-brown structureless area, surrounded by a rim of white scales and a pink structureless area	RPC
10	48	female	chest	pruritus	rheumatoid arthritis treated with azathioprine	keratotic papules with an erythematous border	central yellow-to-brown structureless area surrounded by a thin white irregular rim and pink structureless area	RPC

CRF: Chronic Renal Failure, KD: Kyrle’s Disease, PF: Perforating Folliculitis, RPC: Reactive Perforating Collagenosis.

### 3.2. Literature Review

As previously mentioned, our study also incorporated 19 articles from the literature, comprising 2 case series and 17 case reports, each offering a detailed analysis of the dermoscopic clues and features of APD. Within the analyzed articles, we identified 13 cases of RPC, 6 cases of KD, 3 cases of EPS, and 2 cases of PF. For each study, we documented the author(s), reference numbers, year of publication, study type, demographic data of the included patients, clinical and dermoscopic images, and the final histopathological diagnosis (Table 2). Additionally, we compiled a comprehensive table summarizing the most frequently reported dermoscopic features of PD across all cases in the selected literature (Table 3).

## 4. Discussion

Reactive perforating collagenosis, Kyrle’s disease, elastosis perforans serpiginosa, and perforating folliculitis are uncommon papulonodular dermatoses characterized primarily by the transepidermal elimination of connective tissue components. Due to their shared features, these conditions are often grouped together under the collective term “acquired perforating dermatoses”, highlighting their commonalities over their distinctions.

RPC is reported in the literature as the most common subtype of APD [4]. In our case series, 4 patients were diagnosed with KD, 3 with RPC, and 3 with PF, while no cases of EPS were identified. The median age of our patients (60.7 years) was consistent with previously reported data [26]; however, APD was observed more frequently in women, with 8 out of 10 patients being female. This finding aligns with the observations of Karaali et al. [5] and Edek et al. [27], who also reported a higher prevalence of APD in women. In our study, the lesions were most commonly located on the upper and lower extremities, as frequently documented in the literature [3], and less frequently on the trunk and buttocks. Notably, we identified one case of RPC affecting the ear, which is an uncommon anatomical site for this condition. Pruritus, the most prevalent symptom associated with this condition, was observed in all (10/10) of our patients.

APD typically occurs in patients with various systemic disorders, although its pathogenesis remains poorly understood [1]. In our case series, we reported a patient with microcellular lung cancer undergoing treatment with atezolizumab, carboplatin and etoposide. APD may manifest as a paraneoplastic phenomenon [28] or as a secondary reaction to cancer treatment, particularly immune checkpoint inhibitors [29],; however, such cases are rarely documented in the literature. Pruritus, which occurs as an adverse reaction in nearly 20% of patients receiving checkpoint inhibitors, along with subsequent superficial trauma, is proposed as a primary factor in the development of APD [29].

One of our patients had a recent history of scabies infection and CRF. APD following scabies infection has been previously reported; it is typically seen in conjunction with other comorbidities such as DM and CRF [30]. Ippoliti et al. [30] suggested that the itch–scratch cycle caused by the scabies infection could likely contribute to the development of APD lesions in genetic susceptible individuals.

Lastly, we described two patients receiving azathioprine treatment. Similarly, Grillo et al. [31] reported a case where a patient developed APD three weeks after initiating azathioprine, with symptoms resolving upon discontinuation of the drug. They hypothesized that the immune dysregulation caused by azathioprine might impair fibroblast function in genetically susceptible individuals, leading to focal collagen damage and subsequent elimination of the altered collagen through the epidermis.

**Figure 3:**
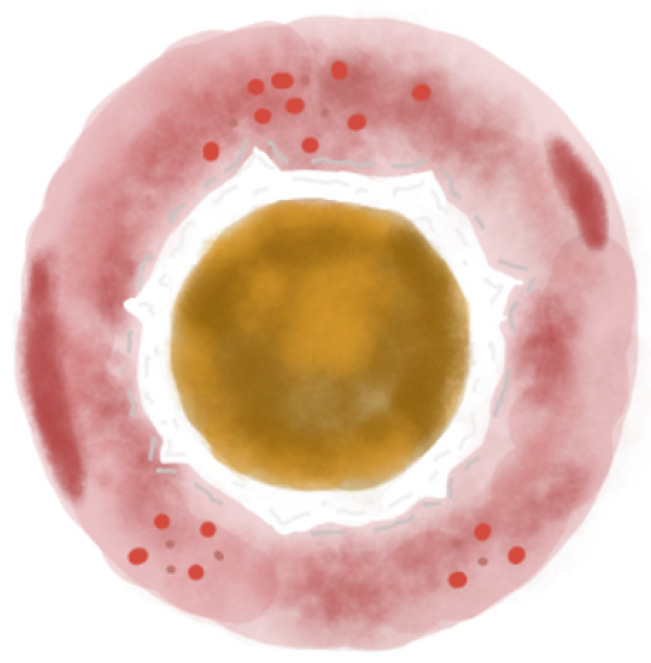
Illustration of the three-zoned concentric pattern, the dermoscopic hallmark of APD. The central zone shows a yellow-to-brown structureless area. The middle zone features a white rim of scales or a broader white structureless area. The outer zone is an erythematous structureless area, occasionally with small vessels, dotted or hairpin. Hemorrhagic spots may also be present.

**Figure 4:**
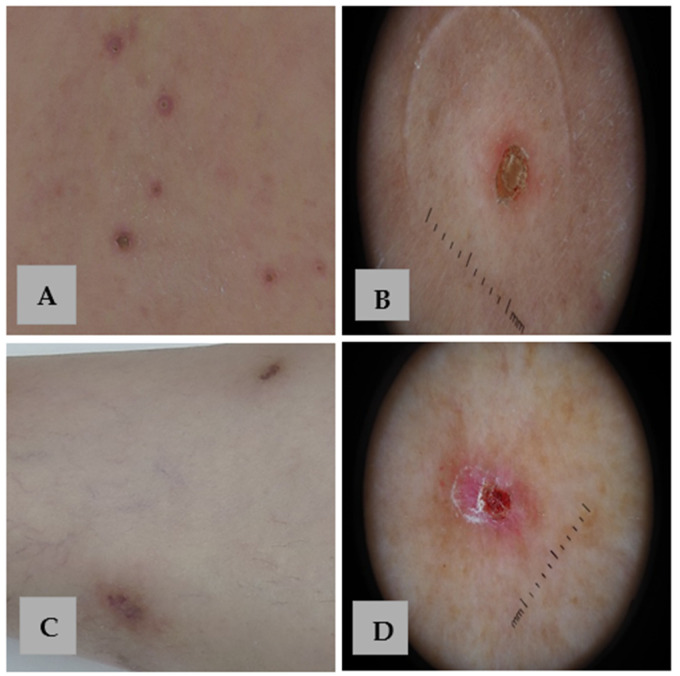
Clinical and dermoscopic comparison of APD and PN. (**A**) Skin-colored and erythematous papules with a central keratotic plug and an erythematous border on the back of a patient with APD. (**B**) Dermoscopy of APD reveals the characteristic concentric pattern. (**C**) Violaceous nodules with a hyperkeratotic surface on the legs of a patient with PN. (**D**) Dermoscopy of PN shows a white “starburst pattern” with radial white lines extending from the center, surrounding hemorrhagic spots and crusts on a red background.

Dermoscopy represents a valuable tool for facilitating earlier diagnosis and providing effective monitoring during the treatment of APD [15]. Nevertheless, the available data on the dermoscopic features of APD remain limited and not fully summarized within the current literature. The concentric pattern appears to be a characteristic feature of APD (Figure 3) and may help clinicians distinguish APD from other similar dermatoses such as PN (Figure 4) [32,33].

Within the central zone of APD lesions, a yellow-to-brown structureless area was commonly observed in our cohort (9/10 patients), consistent with earlier descriptions [1,10,13,15,16,17,18,19,20,21,22,23,24,25], further confirming its diagnostic relevance. Central white clods, previously noted as occasional findings [9,14], were rare in our patients (1/10 patients). In contrast to previous reports describing occasional pink-to-white discoloration and white structureless areas in the center of the lesions [11,12], these features were not observed in our series. These two features were previously described in patients with EPS and may represent dermoscopic clues of this specific subtype of APD. This could account for their absence in our case series, as no patients with EPS were included in our sample. Nevertheless, additional studies involving a larger number of patients are warranted to validate whether these findings are truly specific to EPS.

In the middle zone, the white rim—also referred to as a white “halo”—was reported as the most prevalent feature of this zone in the literature [10,11,14,15,16,20]. This feature was also very frequent in our series (10/10 patients), often accompanied by fine scaling (7/10 patients), which parallels prior observations of surface keratinization [1,8,13,17,20,21,22,23,24]. Conversely, the broader white-to-gray structureless areas described by some authors [8,9,13,14,17,18,19,21,22,25] were not identified in our patients. In certain cases, particularly in PF, coiled-up hair may also be observed [1].

The outer zone, in our case series, typically consisted of an erythematous or erythematous-to-gray structureless area (10/10 patients) indicative of inflammation, a finding consistent with previous studies [8,10,14,15,16,19,20,21,24]. Brown pigmentation and reticular lines, which have been variably described in the literature [9,13,17,18,19,21,22,24,25], were uncommon in our cohort.

Regarding vascular morphology, dotted and hairpin vessels were the predominant vascular structures in our patients (4/10 and 1/10 patients, respectively), in accordance with published data that describe these as the most typical vascular patterns [8,10,16,19,20,21,23,24,25]. Hemorrhagic spots (4/10 patients) and arborizing vessels (0/10 patients), occasionally noted in previous series, refs. [12,21,24] were infrequent in our study.

In the studies conducted by Elmas et al. [34] and Gao et al. [6], it has been suggested that dermoscopic findings may vary depending on the stage of APD disease progression. During the developing stage, dermoscopy typically reveals a yellow-to-brown structureless area, representing a solid central crust that histopathologically corresponds to keratin debris and extruded dermal material. The crust is accompanied by a middle white rim with scaling that corresponds to invaginating epidermal hyperplasia, and a peripheral erythematous area with hairpin, branched, or dotted blood vessels, that reflects a dermal inflammatory reaction with superficial dilated vessels.

In the recovery stage, peripheral blood vessels gradually diminish, replaced by dark-red hyperpigmented areas. The central crust becomes loose and partially desquamated. In the final stage, the central crust detaches, leaving behind a white structureless area in the center, which corresponds histopathologically to fibrosis. This is often accompanied by white scaling and peripheral hyperpigmentation, resulting from the hyperpigmentation of basal keratinocytes.

Behera et al. [35] reported that peripheral hyperpigmentation was a frequent dermoscopic feature in their case series. However, this observation could be explained by the generally darker skin tones within India’s population, as pigmented structures tend to appear more frequently in dermoscopy of a variety of skin lesions in dark-skinned individuals [36].

In certain cases, clinical and dermoscopic evaluations are insufficient to establish a definitive diagnosis. Consequently, histopathological analysis often serves as the diagnostic gold standard. Current studies indicate that more than one subtype of APD can coexist within a single patient. Therefore, it is recommended to perform multiple biopsies to ensure an accurate and comprehensive diagnostic assessment [4,9].

## 5. Conclusions

Our findings align with previously reported dermoscopic features of APD while providing additional insights into the frequency and diagnostic relevance of specific patterns. Larger studies are needed to refine dermoscopic criteria and to identify distinctive features that may help differentiate APD subtypes, thereby improving diagnostic accuracy and patient management.

## Figures and Tables

**Table 2:** Study characteristics.

Authors, Reference Number and Year	Type of Study	Sex/Age (Years)	Distribution	Concomitant Diseases	Clinical Findings	Dermoscopic Findings	Histopathological Diagnosis
Ramirez-Fort et al. [9], 2013	Case report	F/65	Lower extremities	DM, hypothyroidism, dyslipidemia, HT, CRF	Follicular hyperkeratotic, hyperpigmented papules and nodules with central keratotic crust	Central white clod, structureless gray area, brown reticular lines at the periphery	PF
Kittisak P., Tanaka M. [10], 2015	Case report	F/46	Upper and lower extremities	DM, CRF	Ulcerated papules and plaques with central crust	Yellow-to-brown structureless area, white rim, pink erythematous halo, hairpin vessels at the periphery	RPC
Ramirez-Bellver et al. [11], 2015	Case report	F/33	Neck, upper extremities	WD	Keratotic papules in an annular pattern with a depressed yellowish center	Central pink-to- yellow discoloration, brown crusts, white halo	EPS
Navarrete-Dechent et al. [12], 2015	Case report	F/70	Neck, upper extremities, back	DM	Annular erythematous plaques	White structureless area, arborizing vessels	EPS
Russo et al. [13], 2016	Case report	F/38	Upper and lower extremities	N/A	Follicular, keratotic, hyperpigmented papules and nodules	Central crust, scale white-to-gray structureless area, peripheral brown pigmentation	KD
Castrejon-Perez et al. [14], 2016	Case Report	F/64	Face, neck, upper extremities, trunk	HT	Keratotic papules in an annular pattern	Comedo-like structures, gray structureless area, white halo, red background	EPS
Horn et al. [15], 2018	Case series	-M/63 -M/46	-Upper extremities -Upper extremities	-DM, CRF, HT, ischemic heart disease, peripheral vascular disease -DM, CRF, HT	-Keratotic papules and nodules with central keratotic crust -Keratotic papules with central keratotic crust	-Yellow structureless area, white halo, brown-gray structureless area -Yellow-to-brown structureless area, white halo, erythematous-grayish structureless area	-RPC -RPC
Ormerod et al. [16], 2018	Case series	-F/53 -M/67 -M/58 -F/83 -F/80	Trunk and/or lower extremities	-DM, ischemic heart disease, peripheral vascular disease -Pulmonary embolism -Sarcoidosis -Aortic aneurysm, HT, transient ischemic attack, atrial fibrillation -DM, HT, ischemic heart disease	Erythematous keratotic nodules with central keratotic crust in all patients	Yellow-to-brown structureless area, white halo, pink structureless area with dotted and hairpin vessels in all patients	-RPC -RPC -RPC -RPC -RPC
Nair et al. [17], 2018	Case report	M/36	Upper and lower extremities, abdomen	None	Hyperkeratotic, hyperpigmented papules with central keratotic crust	Central crust, scale, gray structureless area, brown pigmentation	KD
Barit et al. [18], 2019	Case report	M/47	Abdomen and extremities	DM, CRF, HT	Keratotic papules with central keratotic plug	Central keratotic plug, white structureless area, peripheral hyperpigmentation	RPC
Wang et al. [19], 2020	Case report	M/50	Trunk and extremities	Alcoholism, Meniere’s disease	Red and brown papules and nodules with central keratotic plug	Central red-brown structureless area, scale, white rim in the middle, red circle with hairpin and dotted vessels at the periphery	RPC
Ozbagcivan et al. [20], 2020	Case report	W/61	Entire body	DM, CRF	Erythematous umbilicated papules with central keratotic plug	Central crust, hemorrhage, scale, white structureless area, structureless pink area with dotted vessels, brown pigmentation	KD
Rajan et al. [21], 2021	Case report	M/11	Abdomen, buttocks, lower extremities	Down’s syndrome, recent femoral shaft fracture	Folliculocentric papules with central keratotic plug	Orange-to-brown clod with coiled- up hair, scale, white-to-gray structureless area	PF
Macca et al. [1], 2023	Case report	M/55	Upper extremities and trunk	CRF, HT	Papules and nodules with central keratotic plug	Central crust, scale, whitish gray structureless area, brown pigmentation at the periphery	KD
Gupta et al. [22], 2024	Case report	M/54	Lower extremities	Hyperuricemia	Erythematous, hyperkeratotic papules and plaques, crateriform erosions, with central keratotic crust	Yellow-to-brown structureless area, scale, linear and dotted vessels, white irregular ring at the periphery	KD
Su et al. [23], 2024	Case report	F/54	Trunk, buttocks, lower extremities	DM, HT, coronary heart disease, uterine fibroid removal	Papules and nodules with central keratotic plug	Yellow-to-brown structureless area, hemorrhage, dotted vessels, scale, pink structureless area, brown pigmentation at the periphery	RPC
Uikey et al. [24], 2024	Case report	F/29	Entire body	Type I DM, HT, pancreatitis, gastric ulcer, hypothyroidism, depression	Papules and nodules with central keratotic plug	Central keratotic plug with a structureless white area, pink structureless area with dotted vessels, structureless brown area at the periphery	KD
Ennaciri et al. [25], 2024	Case report	M/73	Upper and lower extremities	DM	Umbilicated papules and plaques with central keratotic plug	Central crust, scale, white structureless area, pink structureless area with dotted and looped vessels, brown structureless area at the periphery	RPC
Zhang et al. [8], 2025	Case report	M/55	Lower extremities	DM, HT	Erythematous papules and nodules with central keratotic plug and elevated edges	Central brown structureless area, scale, white structureless area, red structureless area with dotted vessels at the periphery	RPC

DM: Diabetes Mellitus, CRF: Chronic Renal Failure, HT: Hypertension, WD: Wilson’s Disease, N/A: Not available.

**Table 3:** Dermoscopic clues of APD according to literature.

Dermoscopic Feature	Lesions, n (%)
**Central area**	
Yellow-to-brown structureless area/crust	20 (83.3)
White clods/comedo-like structures	2 (8.3)
Pink-to-white discoloration	1 (4.1)
White structureless area	1 (4.1)
**Middle zone**	
White rim/halo	11 (45.8)
White/white-to-gray structureless area	10 (41.7)
Scale	10 (41.7)
**Outer zone**	
Erythematous/erythematous-to-gray structureless area	14 (58.3)
Brown pigmentation	9 (37.5)
White-to-gray structureless area	2 (8.3)
Brown reticular lines	1 (4.1)
Crusts	1 (4.1)
**Vessels**	
Dotted vessels	12 (50)
Linear/hairpin vessels	9 (37.5)
Hemorrhage	2 (8.3)
Arborizing vessels	1 (4.1)
**Other features**	
Coiled-up hair	1 (4.1)

## Data Availability

The original contributions presented in this study are included in the article. Further inquiries can be directed to the corresponding author.

## Abbreviations

The following abbreviations are used in this manuscript:

APD	Acquired Perforating Dermatoses
CRF	Chronic Renal Failure
DM	Diabetes Mellitus
EPS	Elastosis Perforans Serpiginosa
HT	Hypertension
KD	Kyrle’s Disease
PD	Perforating Dermatoses
PN	Prurigo Nodularis
PF	Perforating Folliculitis
RPC	Reactive Perforating Dermatosis
WD	Wilson’s Disease

## References

[1] Macca L., Vaccaro F., Li Pomi F., Borgia F., Irrera N., Vaccaro M. (2023). Kyrle disease: A case report and literature review. Eur. Rev. Med. Pharmacol. Sci..

[2] García-Malinis A.J., Del Valle Sánchez E., Sánchez-Salas M.P., Del Prado E., Coscojuela C., Gilaberte Y. (2017). Acquired perforating dermatosis: Clinicopathological study of 31 cases, emphasizing pathogenesis and treatment. J. Eur. Acad. Dermatol. Venereol..

[3] Wagner G., Sachse M.M. (2013). Acquired reactive perforating dermatosis. J. Dtsch. Dermatol. Ges..

[4] Harbaoui S., Litaiem N. (2024). Acquired Perforating Dermatosis. StatPearls [Internet].

[5] Gore Karaali M., Erdil D., Erdemir V.A., Gurel M.S., Koku Aksu A.E., Leblebici C. (2020). Evaluation of clinicopathological and treatment characteristics of 80 patients with acquired perforating dermatosis. Dermatol. Ther..

[6] Gao Z., Lu S.J., Shan S.J. (2023). Acquired perforating dermatosis: A clinicopathologic study, and the features of dermoscopy and reflective confocal microscopy of 37 cases. Ski. Res. Technol..

[7] Rasul T., Wan L., Lawlor A., Cooper B., Khalafbeigi S., Krishnamurthy K. (2025). Kyrle disease: A systematic review of clinical features, diagnostic approaches, dermatoscopic insights, systemic associations, and therapeutic strategies. Arch. Dermatol. Res..

[8] Zhang M., Chu R., Shen J., Liu C., Wang Y., Zhang S., Ren X. (2025). Acquired Reactive Perforating Collagenosis Complicated by Diabetes Mellitus and Hypertension: A Case Report. Case Rep. Dermatol..

[9] Ramirez-Fort M.K., Khan F., Rosendahl C.O., Mercer S.E., Shim-Chang H., Levitt J.O. (2013). Acquired perforating dermatosis: A clinical and dermatoscopic correlation. Dermatol. Online J..

[10] Kittisak P., Tanaka M. (2015). Dermoscopic findings in a case of reactive perforating collagenosis. Dermatol. Pract. Concept..

[11] Ramírez-Bellver J.L., Bernárdez C., Macías E., Moya L., Molina-Ruiz A.M., Cannata Ortiz P., Requena L. (2016). Dermoscopy and direct immunofluorescence findings of elastosis perforans serpiginosa. Clin. Exp. Dermatol..

[12] Navarrete-Dechent C., del Puerto C., Bajaj S., Marghoob A.A., González S., Jaque A. (2015). Dermoscopy of elastosis perforans serpiginosa: A useful tool to distinguish it from granuloma annulare. J. Am. Acad. Dermatol..

[13] Russo T., Piccolo V., Mascolo M., Staibano S., Alfano R., Argenziano G. (2016). Dermoscopy of Kyrle disease. J. Am. Acad. Dermatol..

[14] Castrejón-Pérez A.D., Garza-Chapa J.I., Herz-Ruelas M., Villarreal-Villarreal A., Gomez-Flores M., Gonzalez R., Miran-da-Maldonado L., Ocampo-Candiani J. (2016). Elastosis perforans serpiginosa: Dermoscopic features. J. Am. Acad. Dermatol..

[15] Horn G., Siebel M.d.J.O., Facci D.S., Leda Y.L.d.A., Bet D.L. (2016). Clinical and Dermoscopic correlation of reactive Perforating Collagenosis. Surg. Cosmet. Dermatol..

[16] Ormerod E., Atwan A., Intzedy L., Stone N. (2018). Dermoscopy features of acquired reactive perforating collagenosis: A case series. Dermatol. Pract. Concept..

[17] Nair P., Pariath K. (2018). A Case of Kyrle’s Disease with Dermatoscopic Findings. Indian Dermatol. Online J..

[18] Barit J.-V.J.G., Lizarondo F.P.J., Cubillan E.L.A. (2019). Clinicopathologic and Dermoscopic Features of Acquired Perforating Dermatosis: A Case Report. Acta Med. Philipp..

[19] Wang C., Liu Y.H., Wang Y.X., Zhang J.Z., Jin J. (2020). Acquired reactive perforating collagenosis. Chin. Med. J..

[20] Ozbagcivan O., Lebe B., Fetil E. (2020). Dermoscopic pattern of Kyrle’s disease. An. Bras. Dermatol..

[21] Rajan A., Pai V.V., Shukla P. (2022). Perforating folliculitis in Down’s syndrome—A rare case report. Egypt. J. Dermatol. Venerol..

[22] Gupta S., Gupte R., Manoj R., Raman A., Buccha Y. (2024). An Unusual Case of Kyrle Disease. Cureus.

[23] Su Y., Cui W. (2024). A case report on acquired reactive perforating collagenosis. Medicine.

[24] Uikey D., Thekho A.J., Godara A. (2024). A challenging case of Kyrle’s disease successfully treated with apremilast. Indian J. Dermatol. Venereol. Leprol..

[25] Ennaciri M.A., Damiri A., Khalidi M., Hanafi T., Zemmez Y., El Amraoui M., Frikh R., Hjira N. (2024). Dermoscopy of an acquired perforating dermatosis: A case report. Sch. J. Med. Case Rep..

[26] Karpouzis A., Giatromanolaki A., Sivridis E., Kouskoukis C. (2010). Acquired reactive perforating collagenosis: Current status. J. Dermatol..

[27] Edek Y.C., Aypek Y., Öğüt B., Erdem Ö., Adışen E. (2024). Acquired Perforating Dermatosis: Clinical and Histopathological Analysis of 95 Patients From One Center. Dermatol. Pract. Concept..

[28] Imran N. (2023). Acquired Perforating Dermatosis as a Paraneoplastic Feature: A Case Report, Literature Review, and Novel Association. Case Rep. Nephrol. Dial..

[29] Yamamoto T. (2022). Skin Manifestation Induced by Immune Checkpoint Inhibitors. Clin. Cosmet. Investig. Dermatol..

[30] Ippoliti E., Gori N., Chiricozzi A., Di Stefani A., Peris K. (2024). Scabies-induced perforating dermatosis successfully treated with low dose methotrexate. JEADV Clin. Pract..

[31] Grillo E., Vano-Galván S., Moreno C., Jaén P. (2013). Perforating dermatosis in a patient receiving azathioprine. Indian J. Dermatol..

[32] Wang W., Liao Y., Fu L., Kan B., Peng X., Lu Y. (2021). Dermoscopy Features of Acquired Perforating Dermatosis Among 39 Patients. Front. Med..

[33] Errichetti E., Piccirillo A., Stinco G. (2015). Dermoscopy of prurigo nodularis. J. Dermatol..

[34] Elmas Ö.F., Kilitci A., Uyar B. (2021). Dermoscopic patterns of acquired reactive perforating collagenosis. Dermatol. Pract. Concept..

[35] Behera B., Kumari R., Mohan Thappa D., Hanuman Srinivas B., Gochhait D., Ayyanar P. (2021). Dermoscopic features of acquired perforating dermatosis: A retrospective analysis of 19 cases. Clin. Exp. Dermatol..

[36] Karampinis E., Georgopoulou K.-E., Kampra E., Zafiriou E., Lallas A., Lazaridou E., Apalla Z., Behera B., Errichetti E. (2024). Clinical and Dermoscopic Patterns of Basal Cell Carcinoma and Its Mimickers in Skin of Color: A Practical Summary. Medicina.

